# Detection of Volatile Organic Compounds as Potential Novel Biomarkers for Chorioamnionitis – Proof of Experimental Models

**DOI:** 10.3389/fped.2021.698489

**Published:** 2021-07-22

**Authors:** Sybelle Goedicke-Fritz, Thomas Werner, Hendrik J. Niemarkt, Tim G. A. M. Wolfs, Jörg Ingo Baumbach, Matthew W. Kemp, Alan H. Jobe, Tobias Rogosch, Michelle Bous, Elisabeth Kaiser, Regine Stutz, Sascha Meyer, Rolf Felix Maier, Andreas Rembert Koczulla, Owen Brad Spiller, Boris W. Kramer, Michael Zemlin

**Affiliations:** ^1^Children's Hospital, Philipps University, Marburg, Germany; ^2^Department of General Paediatrics and Neonatology, Saarland University Medical School, Homburg, Germany; ^3^Department of Neonatology, Máxima Medical Centre, Veldhoven, Netherlands; ^4^Department of Pediatrics, School of Oncology and Developmental Biology, Maastricht University, Maastricht, Netherlands; ^5^Center of Competence Breath Analysis, Branch Dortmund, B. Braun Melsungen AG, Melsungen, Germany; ^6^Division of Obstetrics and Gynecology, School of Women's and Infants' Health, University of Western Australia, Crawley, WA, Australia; ^7^Department of Pulmonology, German Center of Lung Research DZL, Institute for Internal Medicine, Philipps-University of Marburg, Marburg, Germany; ^8^Department of Microbiology, School of Medicine, Cardiff University, Cardiff, United Kingdom

**Keywords:** antenatal inflammation, infection, ureaplasma, detection, duration, volatile organic compounds

## Abstract

**Background:** Histologic chorioamnionitis is only diagnosed postnatally which prevents interventions. We hypothesized that volatile organic compounds (VOCs) in the amniotic fluid might be useful biomarkers for chorioamnionitis and that VOC profiles differ between amnionitis of different origins.

**Methods:** Time-mated ewes received intra-amniotic injections of media or saline (controls), or live *Ureaplasma parvum* serovar 3 (Up) 14, 7 or 3d prior to c-section at day 124 gestational age (GA). 100 μg recombinant ovine IL-1α was instilled at 7, 3 or 1d prior to delivery. Headspace VOC profiles were measured from amniotic fluids at birth using ion mobility spectrometer coupled with multi-capillary columns.

**Results:** 127 VOC peaks were identified. 27 VOCs differed between samples from controls and Up- or IL-1α induced amnionitis. The best discrimination between amnionitis by Up vs. IL-1α was reached by 2-methylpentane, with a sensitivity/specificity of 96/95% and a positive predictive value/negative predictive values of 96 and 95%. The concentration of 2-methylpentane in VOCs peaked 7d after intra-amniotic instillation of Up.

**Discussion:** We established a novel method to study headspace VOC profiles of amniotic fluids. VOC profiles may be a useful tool to detect and to assess the duration of amnionitis induced by Up. 2-methylpentane was previously described in the exhalate of women with pre-eclampsia and might be a volatile biomarker for amnionitis. Amniotic fluids analyzed by ion mobility spectrometry coupled with multi-capillary columns may provide bedside diagnosis of amnionitis and understanding inflammatory mechanisms during pregnancy.

## Introduction

Chorioamnionitis (CA) is defined as an acute inflammation of the fetal membranes and amniotic fluids (1–3) and is one of the main causes of preterm delivery and prematurity-associated morbidities worldwide (4–6). Severe cases of CA are associated with maternal complications like sepsis, funisitis and fetal inflammatory response syndrome (FIRS), and neonatal sepsis (7). Chorioamnionitis can result to fetal inflammatory response syndrome that alters the developing immune system and places exposed infants at a higher risk of developing early onset sepsis, bronchopulmonary dysplasia, periventricular leukomalacia and others (8–11).

Clinically chorioamnionitis is diagnosed by the occurrence of maternal fever and two or more of the following: maternal and fetal tachycardia, uterine tenderness, foul smelling amniotic fluid, and maternal leukocytosis (12–20). As a confirmation after delivery, the histologic detection of inflammation and/or microbes in the placenta, amnion, chorion or amniotic fluid are considered the gold standard for diagnosis (21–24). The histologic diagnosis is time consuming. Clinical prediction models for histologic CA have been developed which are however not based on causes of CA (25). Today, different CA laboratory tests are available, but each test has limitations (22, 25). As the early treatment of CA may be dependent on the causative agent. New approaches to improve the rapid diagnosis of CA and the identification of its causing agent is warranted in order to support individualized clinical decision making. Mainly, a screening test should be economical, rapid, non-invasive and specific with respect to the cause of disease. Volatile organic compounds (VOCs) analysis might be a promising new technique for early detection and monitoring of various diseases, including CA (23). VOCs are gaseous carbon-based products of physiologic and pathologic metabolic processes which can be detected in all biological specimens (e.g., breath, urine, feces, blood) (26–30). Recently, multiple proof-of-principle studies have demonstrated the efficacy of using VOC profiles for clinical diagnostics for neonatal sepsis, necrotizing enterocolitis (NEC), and bronchopulmonary dysplasia (BPD) (28–35). This study aimed at identifying VOCs or patterns of VOCs in amniotic fluids that can be used as potential diagnostic markers for amnionitis, e.g., after the discharge of amniotic fluids after premature rupture of membranes. Ion mobility spectrometry (IMS), which allows the detection of VOCs in lower ppb_v_ (parts per billion by volume) to ppt_v_ (parts per trillion by volume) (ng L^−1^ to pg L^−1^) range, have been successfully used for the non-invasive diagnosis of several diseases. Using IMS coupled to multi-capillary columns (MCC/IMS) we have previously established a bedside method that allows the characterization of VOC profiles in the neonatal incubator atmosphere (36).

*Ureaplasma parvum* (Up) is the most prevalent genital Mycoplasma isolated from the amniotic fluid of pregnant women with CA (37). With a colonization rate of 40–80%, Ureaplasma is considered part of the normal genital flora and is transmitted through sexual contact (37, 38). Up can also be transmitted from mother to the fetus or at the time of birth (3, 39). Among multiple inflammatory cytokines and chemokines that are increased in amniotic fluid during CA, interleukin-1 alpha (IL-1α) has been identified to play a key role in the progression of preterm labor and of the fetal inflammatory response (40, 41). IL-1α is induced by many different bacteria or bacterial components and can thus be considered a part of the common effector mechanism of bacterial infection, including amnionitis with Up (42). We compared a clinical chorioamnionitis model, i.e., Up, with the common endpoint of bacterial activation, i.e., IL-1α, to distinguish between changes induced by the Up bacteria in comparison to changes induced by the inflammation as such.

Using a well-established pre-clinical model of chorioamnionitis, the aim of his study was to demonstrate that VOC profiles can be measured from amniotic fluids using MCC/IMS. We compared the time course of VOC profiles of amniotic fluids in pregnant sheep with Up induced or IL-1α- induced CA.

## Methods

The animal study and experimental protocols were carried out in accordance with the guidelines “Animal Welfare Act (2002)” and the “Australian code for the care and use of animals for scientific purposes (8th edition, 2013).” All animal experiments were approved by the Animal Ethics Committee of the University of Western Australia (reference number RA/3/100/312) and were performed at The University of Western Australia (Perth, Australia). This study was approved by the Animal Ethics Committees at the Children's Hospital Medical Center in Cincinnati and Western Australian Department of Agriculture.

Preparation of recombinant ovine Interleukin-1α (IL-1α) was performed as previously described (40). Briefly, IL-1α were cloned into the vector pET-30 Xa/LIC (Novagen, Madison, WI), custom expressed (Protein Express, Cincinnati, OH) in *E. coli* BL21 (DE3), purified by metal chelate chromatography and cleaved with Factor Xa. Recombinant IL-1α were collected using Ni-NTA columns and purified by gel filtration chromatography/ and cation exchange chromatography, respectively, followed by removal of endotoxin by passage over polymyxin B agarose columns (Pierce, Rockford, IL).

**Figure 1:**
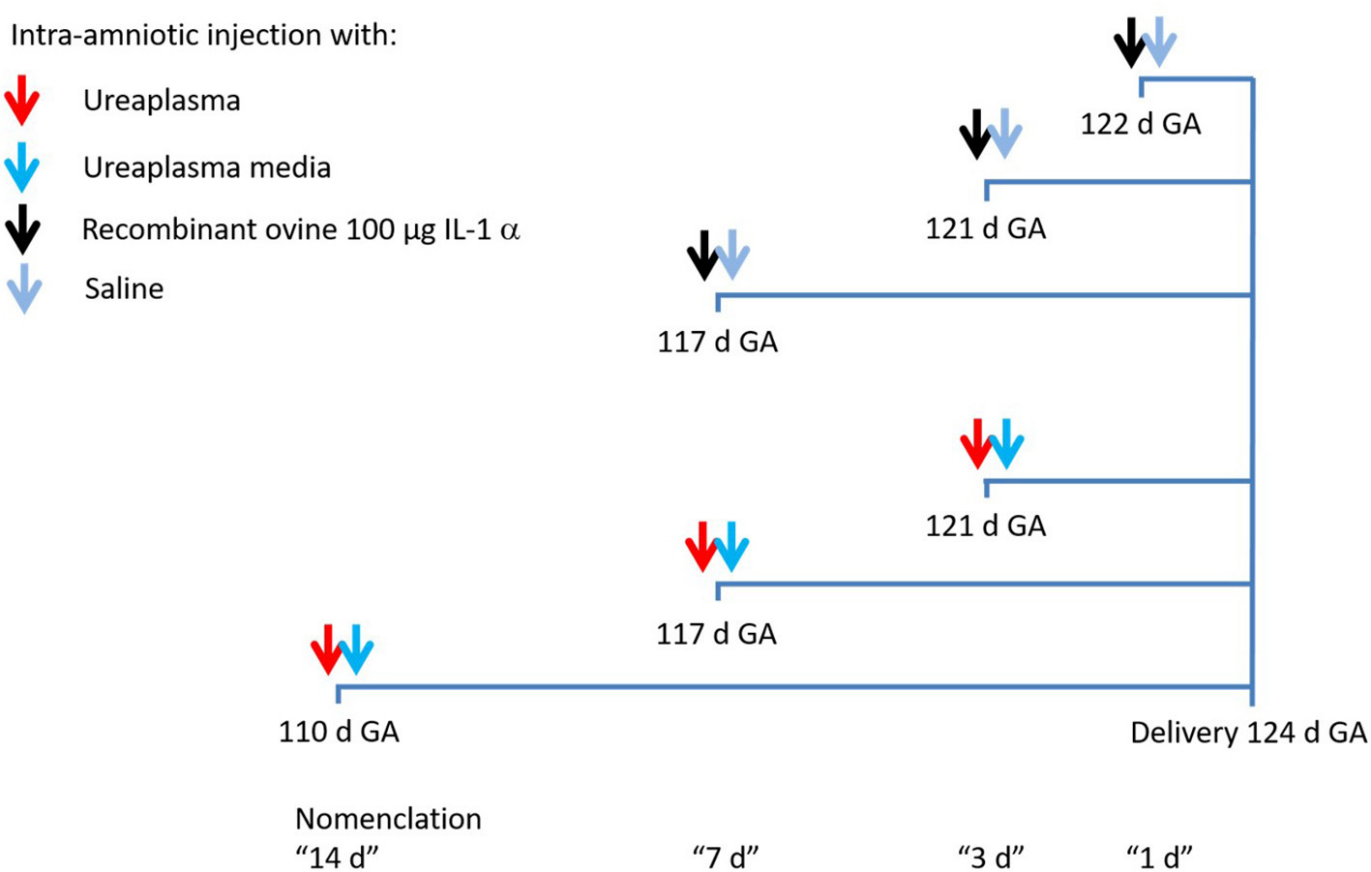
Experimental design. Antenatal inflammation was induced by a single injection of IL-1α under ultrasound guidance at 117, 121 or 122 d gestational age (GA) or *Ureaplasma parvum* at 110, 117 or 121 d GA. Animals were delivered at 124 d GA and animals of the control group underwent the same procedure with an injection of saline or *Ureaplasma* media.

*Ureaplasma parvum serovar 3* was isolated as previously described (43). Fetal treatments were performed as previously described (44). 53 ewes received intra-amniotic (IA) injections of media (control) (*n* = 6) or live Ureaplasma (*Ureaplasma parvum; strain HPA5, serovar 3*) at 14 (*n* = 5), 7 (*n* = 11) or 3 (*n* = 12) days (d) or recombinant bovine IL-1α at 7 (*n* = 7), 3 (*n* = 5) or 1 (*n* = 7) days before delivery at 124 d GA (Figure 1). The time points were chosen based on previous results on the kinetics of Up induced changes in lung and gut and the fast response to recombinant IL-1 alpha (41, 43). During surgical delivery amniotic fluid samples were sterilely collected, aliquoted, immediately snap frozen and stored at −80°C until analysis. Chorioamnionitis was confirmed by histologic analysis in all *Ureaplasma parvum* and IL-1α injected ewes as previously described (41, 43).

VOCs were detected as head space measurements using multi-capillary column ion mobility spectroscopy Breath Discovery system (MCC/IMS) (B&S Analytik GmbH, Dortmund, Germany). Isothermal pre-separation was performed at 40°C with an OV-5 multi-capillary column (MCC) (Multichrom, Novosibirsk, Russia) at a flow of 150 mL/min. During sample collection, the drift flow was set to 100 mL/min and the ions were detected in positive mode. The collection time was 20 seconds. The device and sampling parameters are given in Supplementary Table 1. The methods for VOC analysis were published earlier (36). A laboratory bottle (100 mL) heated to 37°C served as a sample container. A closed system was established: a large laboratory bottle (1000 mL, gas reservoir, Schott Duran®, Wertheim/Main, Germany) was connected to the small bottle (100 mL, Schott Duran®, Wertheim/Main, Germany) via a perfluoroalkoxyalkane (PFA) tube that was led through the caps. Both bottles were filled with synthetic air as carrier gas. Another tube connected the cap of the small laboratory bottle and the Sampling Input of MCC/IMS device. Synthetic air was used to exclude contamination with environmental VOCs as previously published (36).

### Statistical Evaluation

The MCC/IMS data were evaluated using the software Visual Now 2.5 (B & S Analytik, Dortmund Germany) as previously reported (36). All peaks were characterized by their specific combination of position retention time per second and drift time (corresponding 1/K0-value) (45–49). The height of the peak was a measure for the concentration of the VOCs. The databank layer 20160426_SubstanzDbNIST_122_St_layer (B & S Analytik GmbH, 2016) was used for peak referencing and determination of retention times and 1/K0-values according to Steinbach et al. (36). Box-and-Whisker plots and a rank sum test (Wilcoxon-Mann-Whitney test using Bonferroni correction) were used to identify significant differences between sample groups. Significant peaks (*p* < 0.05) were used for further evaluation with decision trees (DT) using RapidMiner Studio Free 8.2.001 (RapidMiner GmbH, Dortmund, Germany) (36).

## Results

**Figure 2:**
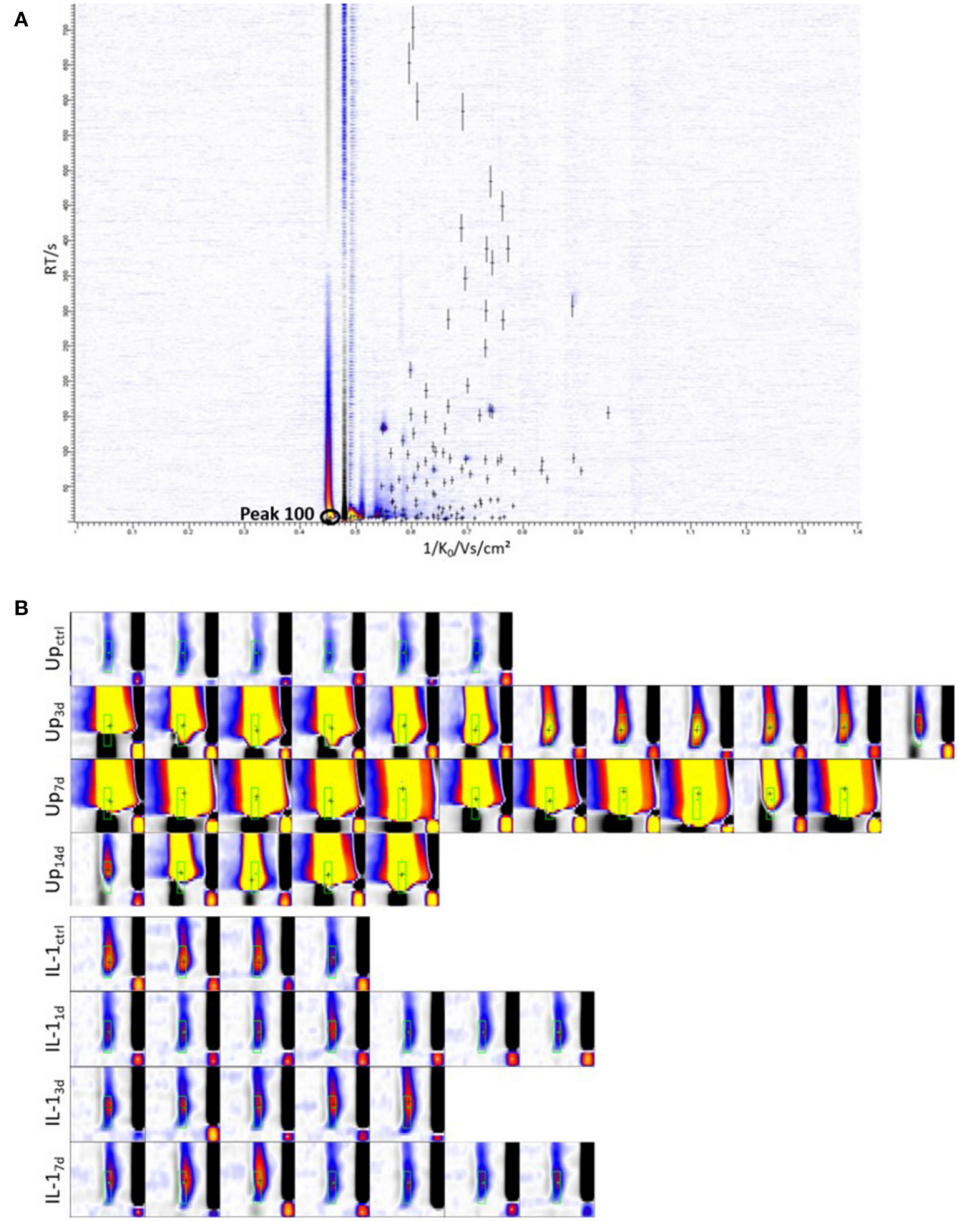
Representative chromatogram and intensity distribution of peak 100 (2-methylpentane). **(A)** All 127 peaks identified in amniotic fluid are indicated with crosses. The y-axis of the heat map represent the retention time and x-axis represent 1/K0, a transformation of the drift time. The colors display the signal intensities with increasing values from white over blue and red to yellow. **(B)** Shown are the cutouts of MCC/IMS-Chromatograms of peak 100 for every single measurement. White = no signal, blue = low signal, red = medium signal, yellow = high signal, black = reactant ion peak (RIP), x- axis = inverse mobility 1/K_0_/Vs/cm^2^. Up*, Ureaplasma parvum*; IL-1, Interleukin-1α.

A total of 127 signals (peaks) were identified, as characterized by drift and retention time of ions formed from amniotic fluids of sheep after intra-amniotic instillation of *Ureaplasma parvum*, Interleukin-1α samples and controls (Figure 2A, Supplementary Table 1).

**Figure 3:**
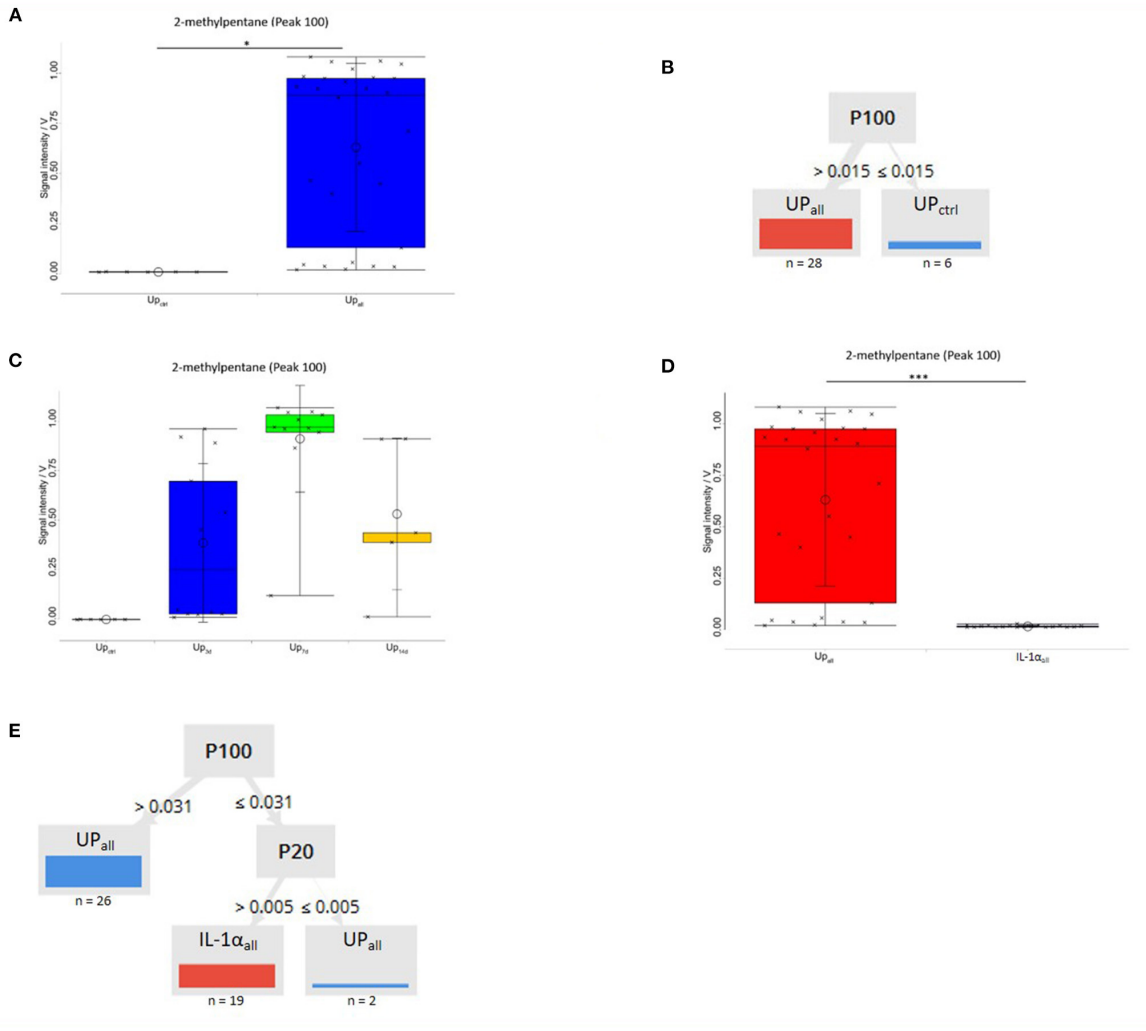
Box-and-Whisker Plot of peak 100 (2-methylpentane) of all samples medium vs. all samples Up. **(A)** 2-methylpentane was significantly higher in Up all samples vs. all samples medium control (Up_ctrl_ *n* = 6, Up_all_ *n* = 28). Sensitivity/specificity was 100/100% with positive predictive value/negative predictive value 100/100%. Measurements are marked with crosses. Significance levels after Bonferroni correction are indicated (*p* < 0.05). **(B)** A decision tree based on one compounds is shown. Samples are grouped according to the means of the peak intensity of each compound, at which point, the maximum number of samples are classified correctly. Relative numbers of classified all samples medium control (Up_ctrl_) are blue, and numbers of classified all samples Up (Up_all_) are red. Discrimination of the two groups is possible. Sensitivity/specificity was 100.0/100.0% with positive predictive value/negative predictive value 100.0/100.0%. Every measurement was correctly assigned to its class. **(C)** 2-methylpentane concentrations were significantly elevated compared to controls at 3 days post-infection (Up_ctrl_ *n* = 6, Up_3d_ *n* = 12; Up_ctrl_ vs. Up_3d_ *p* < 0.001), appeared to peak at 7 Day post-infection (Up_ctrl_ *n* = 6, Up_7d_ *n* = 11; Up_ctrl_ vs. Up_7d_ *p* < 0.001) and then appeared to be declining in intensity by 14 post-infection (Up_ctrl_ *n* = 6, Up_14d_ *n* = 5; Up_ctrl_ vs. Up_14d_ *p* < 0.01). Sensitivity/specificity were 100/100% with positive predictive value/negative predictive value 100/100% for each comparison. **(D)** Box-and-Whisker Plot of best separating peak 100 (2-methylpentane) between Up and IL-1α. 2-methylpentane was significantly higher in all samples Up vs. all samples IL-1α (Up_all_ *n* = 28, IL-1α *n* = 19). ^***^p < 0.001 after Bonferroni correction. **(E)** Decision tree algorithm to discriminate between all samples Up and all samples IL-1α. Discrimination of the two groups is possible by using peak 100 (2-methylpentane) and peak 20. ^*^p < 0.05 after Bonferroni correction.

Comparing all control samples (injected with sterile medium) to samples from Up–infected animals identified 27 substances that differed significantly (Up_ctrl_ *n* = 6, Up_all_ *n* = 28; *p* < 0.05). After Bonferroni *post-hoc* analysis correction, one single peak reached a significance of *p* < 0.05 for the distinction of medium controls vs. Up (Figures 2B, 3A). With the BS-MCC/IMS-analyses database (Version 1209), this compound was identified as 2-methylpentane (isohexane) according to the retention time and drift time. The average signal intensities of 2-methylpentane was higher for Up samples than for medium samples (Up_ctrl_ *n* = 6, Up_all_ *n* = 28) (Figures 2B, 3A). For 2-methylpentane the sensitivity and specificity for Up CA was 100%, respectively, and both the positive and negative predictive values were 100%, respectively (Table 1). The decision tree achieved a 100% separation of medium and Up samples using 2-methylpentane in the data set (Figure 3B).

**Table 1:** Statistical analyses for peak 100 (2-methyl-pentane).

**Best direction**	**Medium < Up_**all**_**	**Up_all_ > IL-1α_all_**
Best threshold	0.020	0.022
Classified right	34	45
Classified wrong	0	2
True positive	6	27
False positive	0	1
True negative	28	18
False negative	0	1
Sensitivity %	100	96
Specificity %	100	95
Positive predictive value %	100	96
Negative predictive value %	100	95
Accuracy %	100	96
Significance^*^	<0.05	<0.001

* *Significance after Bonferroni correction*.

In the next step we studied 2-methylpentane in amniotic fluids 3, 7, and 14 days after the intra-amniotic instillation of Up (Up_3_ *n* = 12, Up_7_ *n* = 11, Up_14_ *n* = 5). 2-methylpentane was present at all time points and after short term exposure to Up (Figure 3C). Moreover, 2-methylpentane signal intensities were more variable, yet still significantly elevated compared to controls at 3 days post-infection (Up_ctrl_ vs. Up_3_ *p* < 0.001; Up_ctrl_ *n* = 6, Up_3_ *n* = 12), appeared to peak at 7 days post-infection (with a much less variation between infected samples; Up_ctrl_ vs. Up_7_ *p* < 0.001; Up_ctrl_ *n* = 6, Up_7_ *n* = 11) and then appeared to be declining in intensity by 14 post-infection (Up_ctrl_ vs. Up_14_ *p* < 0.01; Up_ctrl_ *n* = 6, Up_14_ *n* = 5) (Figure 3C). To clarify whether 2-methylpentane was a result of infection with Up or IL-1α induced inflammation, we compared all samples of Up induced CA with samples of IL-1α induced CA (Up_all_ *n* = 6; IL-1α *n* = 19). Direct comparison resulted in 9 peaks that differed significantly after Bonferroni *post-hoc* analysis correction (*p* < 0.05). Interestingly, 2-methylpentane levels differed significantly after Bonferroni correction at a level of *p* < 0.001 between all samples with Up vs. all samples with IL-1α and reached a sensitivity/specificity of 96/95% with positive predictive value/negative predictive values of 96 and 95%, respectively (Figure 3D). The average relative signal intensities for 2-methylpentane were higher for Up samples than for IL-1α samples. The decision tree reached a 100% separation of Up and IL-1α samples using 2-methylpentane and P20 (Figure 3E). The comparison of all IL-1α samples against saline controls and of the individual time points against saline controls, none significant difference was detected (data not shown).

## Discussion

In this study we demonstrate that the measurement of VOC profiles in amniotic fluids by ion mobility spectrometry may have a diagnostic potential for CA. With this novel technique, it was possible to differentiate VOC profiles in CA of different origin (Up vs. IL-1α induced CA) and to estimate the duration of the inflammation in an experimental sheep model. This strategy could be useful to develop a rapid bedside test for detecting cause specific CA using amniotic fluids.

We designed the study to differentiate between the most common bacteria isolated from CA and an essential signaling pathway induced by bacteria. With this approach we were able to distinguish between healthy controls and CA and even to differentiate between CA induced by an UP in comparison to CA induced by IL-1α. Therefore, we searched for VOCs that allowed to differentiate between a bacterial cause vs. a sterile cause for similar histologic endpoints. The common histologic endpoint of CA usually does not allow distinguishing between various causes of infection.

All organisms produce VOCs as part of their normal metabolism, and it has long been known that certain infections are accompanied by a distinct odor (50, 51). Breathomics has been used to study volatile organic compounds produced by certain species or strains of bacteria in septic patients (52). Fetid amniotic fluids are a hallmark of amnionitis, indicating that particular VOCs might be set free that could be detected by MCC/IMS. To test this hypothesis, we have developed a head space method that allows VOC measurements independently of the ambient air or other exogenous influences.

During an infection, VOCs can originate from the host or from the infectious organism itself. The biomarkers of inflammation which are most increased in the human body are ammonia, acetone, isoprene, nitic oxide, hydrogen sulphide, methane, ethane and pentane (53). On the other hand, pathogens can produce a wide variety of VOCs, including various fatty acids and their derivatives including hydrocarbons, alcohols and ketones (53).

In this study we used a validated Up induced CA model in sheep. It was confirmed that the infection with Up was associated with changes in the VOC profile within the amniotic fluid, using medium samples as control. Reassuringly, the average signal intensity of 2-methylpentane was higher for Up samples than for medium samples. The measured sensitivity, specificity as well as positive and negative predictive values of 100% were remarkable. Moreover, the relative concentration of 2-menthylpentane varied with the time course of CA. The signal strength was highest on day 7. 2-methylpentane was only detected in amniotic fluids after infection with Up and not in IL-1α induced CA. We therefore hypothesize that 2-methylpentane was associated with Up infection in a time-dependent manner and independent of the IL-1α mediated inflammatory pathway.

2-methylpentane belongs to the group of pentanes which are produced by the oxidation of cellular lipids (54). In exhaled breath, pentanes can be indicative of oxidative stress, physical and mental stress, arthritis, breast cancer, asthma, chronic obstructive pulmonary disease (COPD), inflammatory bowel diseases, sleep apnea, ischemic heart disease, myocardial infarction, liver disease, schizophrenia, sepsis and other conditions (55–61). Interestingly, 2-methylpentane has been previously identified in breath condensates from pregnant women suffering from pre-eclampsia and in preterm neonates developing BPD (61). BPD is associated with Ureaplasma infection (62, 63). However, biomarkers of oxidative stress such as breath pentanes are inherently non-specific markers of disease because they are increased in a wide variety of conditions. Therefore, we cannot further pinpoint the detection of 2-methylpentane to the specific pathway of the bacterial or human metabolism. Although 2-methylpentane was only observed in the presence of a living micro-organism, the intensity of the peak did not correlate with the colony forming units within the amniotic fluids (data not shown) and its origin remains unknown. In example, the source of 2-methylpentane could be Up, the mother, or the fetus.

Chorioamnionitis is a major cause of premature rupture of membranes and of preterm birth and can be difficult to diagnose. Current diagnostic strategies mainly rely on clinical signs and laboratory test in maternal blood. However, amniotic fluids can be accessible upon premature rupture of membranes and during diagnostic amniocentesis.

Strengths of the current study are the controlled environment of the established model that minimizes confounding factors including behavioral and dietary influences and the use of a precisely regulated amount of Up, which are the most common microorganisms isolated in cases of spontaneous preterm birth. Our study is limited to a single pathogen. Therefore, further studies should be performed to determine VOC profiles in CA of different origins (64). Additional limitations include small sample sizes which are inherent in large mammalian animal models. Also the samples obtained by amniocentesis were examined and in further studies it must be studied whether these differences can also be detected in amniotic fluids that are discharged vaginally after premature rupture of membranes. Thus future studies are required to identify VOCs with a diagnostic potential in amniotic fluids of humans.

In conclusion, measurements with ion mobility spectrometry coupled with multi-capillary columns indicate that volatile organic compounds may be a useful tool to detect CA and to distinguish between CA of differing origins (Up vs. IL-1α induced CA in sheep) and to estimate the duration of the inflammation. We have identified 2-methylpentane as a potential volatile organic compounds that might be a useful biomarker for Up-induced CA. Thus, our results might serve as a proof of principle study to use VOCs in amniotic fluids as a rapid bedside test to improve the timely diagnosis of amnionitis.

## Data Availability Statement

The raw data supporting the conclusions of this article will be made available by the authors, without undue reservation.

## Ethics Statement

The animal study was reviewed and approved by Animal Ethics Committee of the University of Western Australia (Reference Number RA/3/100/312).

## Author Contributions

SG-F conception and design, collection and/or assembly of data, data analysis and interpretation, manuscript writing, final approval of manuscript. TW collection and/or assembly of data, final approval of manuscript. HN, TGW, MK, AJ, and TR provision of study material or patients, final approval of manuscript. MB, EK, RS, SM, and AK manuscript writing, final approval of manuscript. JB data analysis and interpretation, manuscript writing, final approval of manuscript. BK conception and design, provision of study material or patients, data analysis and interpretation, manuscript writing, final approval of manuscript. MZ and RM conception and design, data analysis and interpretation, financial support, manuscript writing, final approval of manuscript. All authors have read and agreed to the published version of the manuscript.

## Conflict of Interest

TR is an employee of CSL Behring. JB is an employee of B. Braun Melsungen AG. The remaining authors declare that the research was conducted in the absence of any commercial or financial relationships that could be construed as a potential conflict of interest.

## Abbreviations

BPD: Bronchopulmonary dysplasia
CA: Chorioamnionitis
D: Days
DT: Decision tree
FIRS: Fetal inflammatory response syndrome
GA: Gestational age
IMS: Ion mobility spectrometry
IL-1α: Interleukin-1 alpha
MCC: Multi-capillary column
MCC/IMS: Ion mobility spectrometer coupled to multi-capillary column
NEC: Necrotizing enterocolitis
NI: Neonatal incubators
PCA: Principal Component Analysis
PFA: Perfluoroalkoxyalkane
RIP: Reactant ion peak
Up: *Ureaplasma parvum* serovar 3
VOC: Volatile organic compound.
