# Supplementary material for: Detection of Volatile Organic Compounds as Potential Novel Biomarkers for Chorioamnionitis – Proof of Experimental Models
Source: Front Pediatr. 2021 Jul 22;9:698489. doi: 10.3389/fped.2021.698489 (PMC8339372; doi:10.3389/fped.2021.698489)
Supplement: Supplementary file 1 [file Data_Sheet_1.PDF]

Supplementary Material

1 Supplementary Tables

Supplementary Table 1: Device and sampling parameters.

**Carrier gas (Ionisation)**	63Ni (95 MBq)
**Column Ion separation Drift tube**	330 V /cm, 0.12 m
**Column temperature**	40 °C
**Detection mode**	positive
**Drift flow**	100 mL /min
**MCC Flow**	150 mL /min
**Multi Capillary Column (MCC)**	OV-5
**Sample Flow**	100 mL /min
**Sampling duration**	20 s
**Synthetic air**	20.5 ± 0.5 % O2 in N2, purity ≥ 99,999 mol %)

Supplementary Table 2: Peaks were characterized by their specific combination of position retention time (RT) per second and drift time (corresponding 1/K0-value)

Name	1/K0	RT
P0	0,549	132,6
P1	0,599	216,9
P2	0,604	125,7
P3	0,584	116,2
P4	0,557	9,0
P5	0,542	6,9
P6	0,590	4,9
P7	0,608	23,9
P8	0,611	5,5
P9	0,660	4,7
P10	0,681	4,9

P11	0,648	4,9
P12	0,697	89,2
P13	0,565	45,4
P14	0,529	7,4
P15	0,572	20,5
P16	0,567	29,4
P17	0,539	9,0
P18	0,672	9,0
P19	0,583	9,9
P20	0,611	9,0
P21	0,648	9,4
P22	0,554	4,5
P23	0,661	3,1
P24	0,565	50,0
P25	0,549	9,6
P26	0,549	2,6
P27	0,582	2,6
P28	0,639	10,0
P29	0,670	20,6
P30	0,763	449,4
P31	0,714	23,0
P32	0,783	23,0
P33	0,745	156,8
P34	0,889	307,4
P35	0,626	149,3
P36	0,689	5,6
P37	0,536	6,0
P38	0,523	6,0
P39	0,505	6,0
P40	0,573	6,0

P41	0,629	5,6
P42	0,714	5,6
P43	0,664	4,9
P44	0,640	6,0
P45	0,607	6,0
P46	0,745	6,0
P47	0,758	5,0
P48	0,692	6,6
P49	0,570	13,0
P50	0,511	12,0
P51	0,492	8,4
P52	0,490	5,0
P53	0,509	4,0
P54	0,766	7,4
P55	0,545	14,5
P56	0,514	6,5
P57	0,497	8,0
P58	0,628	55,9
P59	0,625	86,5
P60	0,612	78,9
P61	0,649	20,5
P62	0,565	27,5
P63	0,546	19,0
P64	0,554	15,9
P65	0,585	17,5
P66	0,611	598,5
P67	0,666	164,8
P68	0,689	418,9
P69	0,722	152,3
P70	0,706	67,9
P71	0,599	153,3

P72	0,692	584,1
P73	0,659	55,9
P74	0,563	98,4
P75	0,696	346,9
P76	0,745	369,0
P77	0,578	60,4
P78	0,833	73,4
P79	0,891	90,4
P80	0,773	388,8
P81	0,639	40,5
P82	0,547	51,5
P83	0,741	159,2
P84	0,660	133,4
P85	0,626	187,1
P86	0,734	300,8
P87	0,673	59,4
P88	0,642	39,4
P89	0,639	107,3
P90	0,593	48,4
P91	0,640	75,2
P92	0,733	247,4
P93	0,701	194,1
P94	0,666	288,3
P95	0,606	63,3
P96	0,690	75,8
P97	0,669	90,7
P98	0,733	88,7
P99	0,761	90,2
P100	0,454	6,0
P101	0,596	653,4

P102	0,764	287,5
P103	0,655	13,1
P104	0,618	11,6
P105	0,588	11,1
P106	0,682	12,0
P107	0,643	99,9
P108	0,756	86,5
P109	0,834	86,0
P110	0,755	31,6
P111	0,742	31,6
P112	0,723	30,5
P113	0,725	25,0
P114	0,692	19,5
P115	0,608	31,1
P116	0,657	19,0
P117	0,736	61,0
P118	0,784	72,4
P119	0,594	96,0
P120	0,657	98,9
P121	0,603	703,5
P122	0,953	155,8
P123	0,905	72,4
P124	0,843	61,0
P125	0,742	484,8
P126	0,735	388,5
